# Supplementary material for: The impact of multidomain interventions on cognitive and physical function in older adults with subjective cognitive decline: a meta‑analysis and systematic review
Source: PeerJ. 2025 Jun 26;13:e19588. doi: 10.7717/peerj.19588 (PMC12206402; doi:10.7717/peerj.19588)
Supplement: Supplemental Information 1 [file peerj-13-19588-s001.docx]

**Combination Search Terms**

This study have searched the four databases: PubMed, Embase, Cochrane Library and Web of Science.

The databases were retrieved using the following combination methods:(( #1 OR (#2 AND #3)) AND #4 AND #4

| **Classifications** | **Specific terms** |
| --- | --- |
| **#1 Multi-domain interventions** | "multidomain intervention*" OR "multi-domain intervention*" OR "multicomponent intervention*" OR "dual task" OR "life style intervention*" OR "interdisciplinary intervention" OR multidomain* OR multicomponent* OR multidisciplin* OR "multifactorial intervention*" OR "multi-factorial intervention" OR "combined intervention*" OR "multiple component intervention*" OR "multi-dimensional intervention*" |
| **#2 Nutritional intervention terms** | nutrition OR "nutrition intervention*" OR "nutrition therapy" OR "nutritional assistance" OR "nutrition supplement" OR micronutrients OR macronutrients OR diet* OR dietary OR "dietary intervention*" OR "dietary supplement*" OR "diet therapy" OR "lifestyle intervention*" OR "Mediterranean Diet" OR "Ketogenic diet" OR "DASH diet" OR "Atkins diet" OR "carbohydrate-restricted" OR "Caloric restriction" OR "fat-restricted" OR "Weight reduction programs" OR "weight intervention" |
| **#3 Exercise intervention terms** | exercis* OR sport* OR "physical activity" OR "physical exercise" OR "physical training" OR "sports training" OR "aerobic exercis*" OR "aerobic train*" OR "aerobic fitness" OR "aerobic program*" OR "resistance exercis*" OR "resistance train*" OR "anaerobic exercis*" OR "anaerobic train*" OR "resistance program*" OR movement OR "functional training" OR "core training" OR "acute exercise*" OR "isometric exercises" OR "flexibility training" OR "high-intensity interval training" OR HIIT OR walking OR resistance OR strength OR bicycl* OR "bike rid*" OR "bicycle rid*" |
| **#4 cognitive impairment** | "subjective cognitive decline" OR "subjective memory impairment*" OR "subjective memory decline" OR "subjective memory complaints" OR "subjective cognitive dysfunction" OR "subjective cognitive impairment*" OR SCD OR "memory decline" OR "cognitive decline" |
| **#5 Aging population terms** | "older adults" OR elder* OR senior* OR adult* OR older OR aging OR "the aged" OR "old people" OR "senior citizens" OR retirement |
