# Supplementary figures and images for: The impact of multidomain interventions on cognitive and physical function in older adults with subjective cognitive decline: a meta‑analysis and systematic review
Source: PeerJ. 2025 Jun 26;13:e19588. doi: 10.7717/peerj.19588 (PMC12206402; doi:10.7717/peerj.19588)

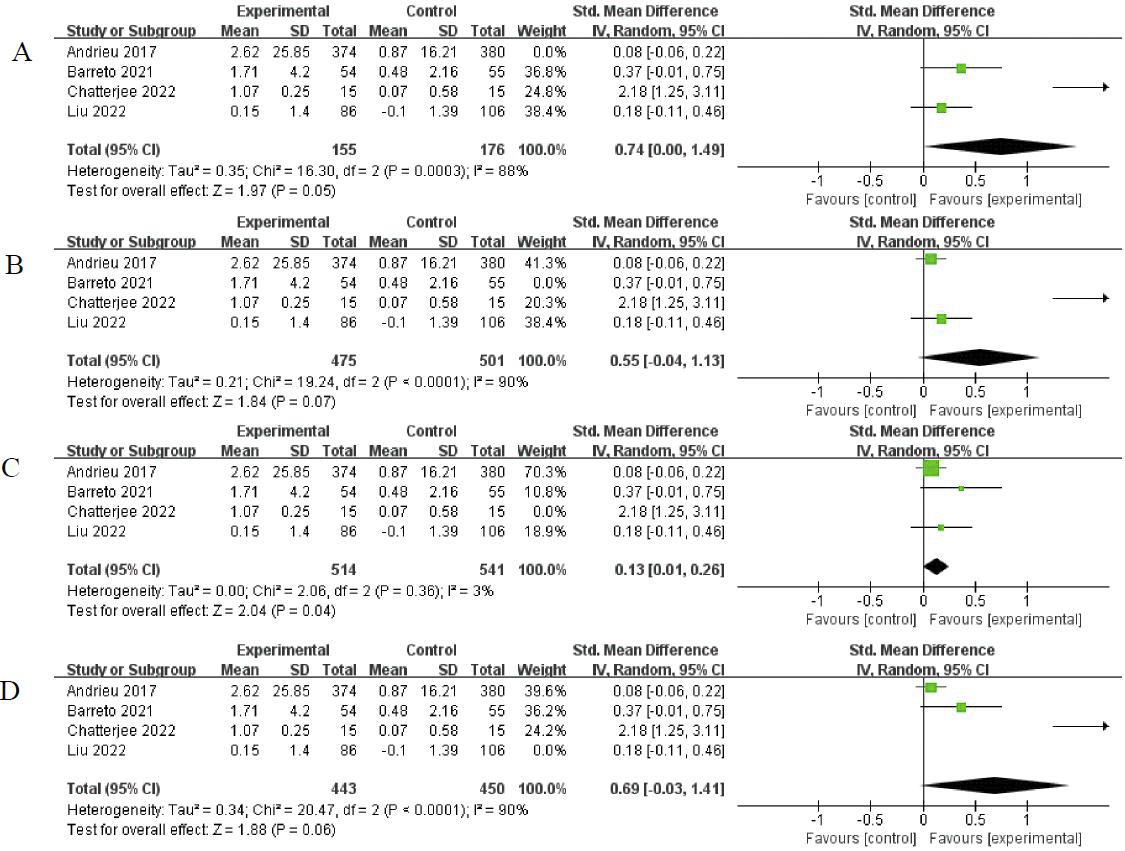

Supplement: Supplemental Information 2 [file peerj-13-19588-s002.tif]

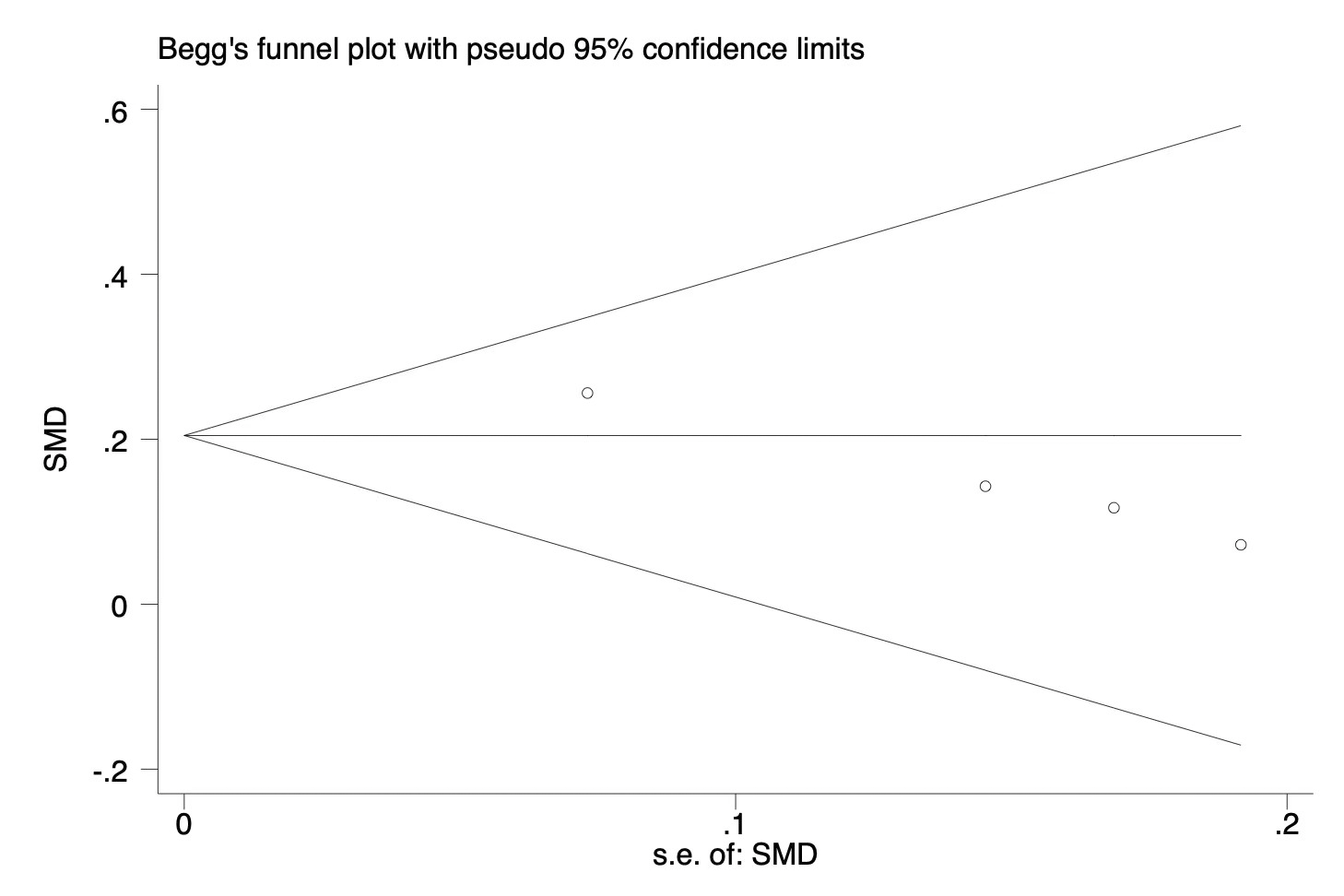

Supplement: Supplemental Information 3 [file peerj-13-19588-s003.tif]

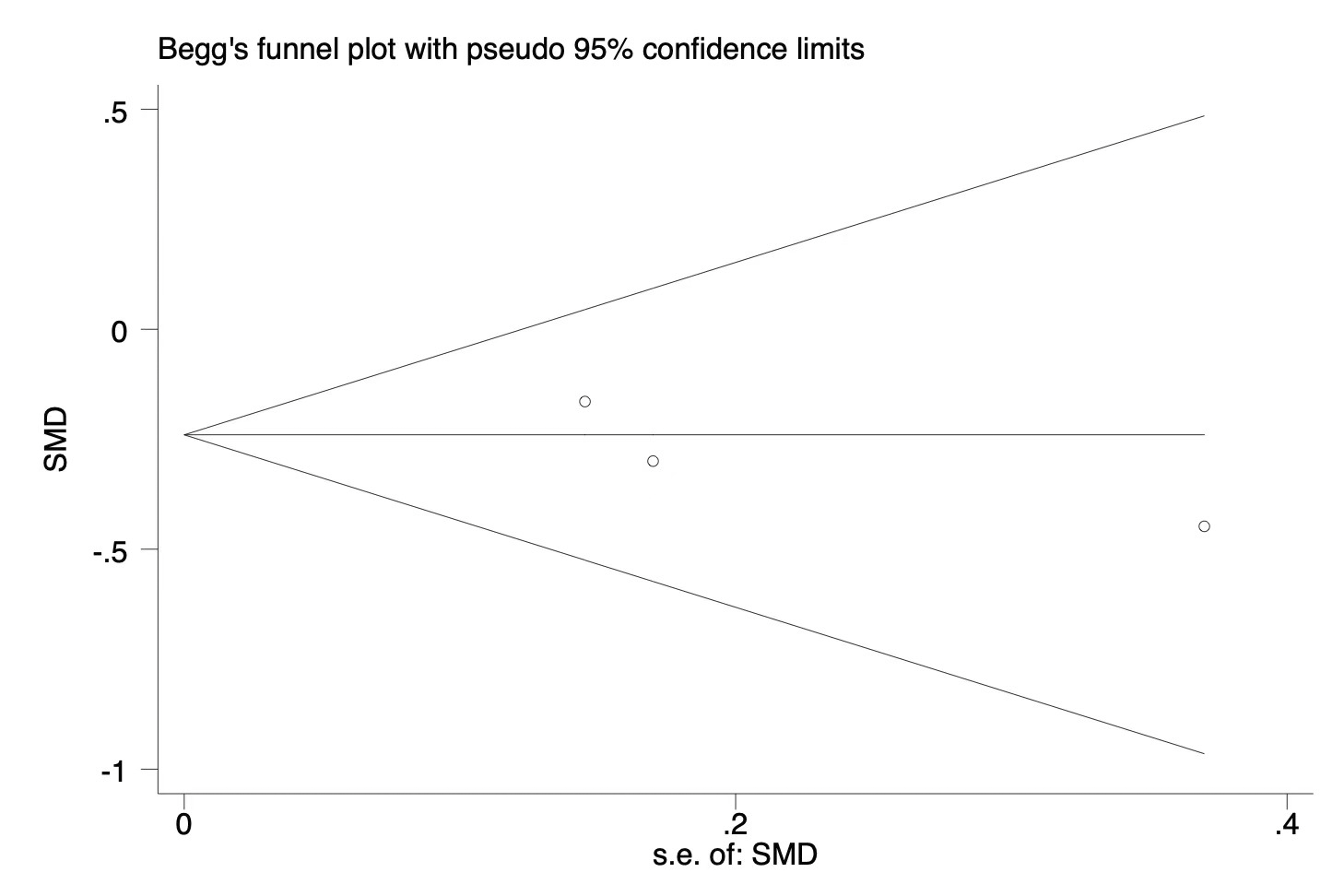

Supplement: Supplemental Information 4 [file peerj-13-19588-s004.tif]

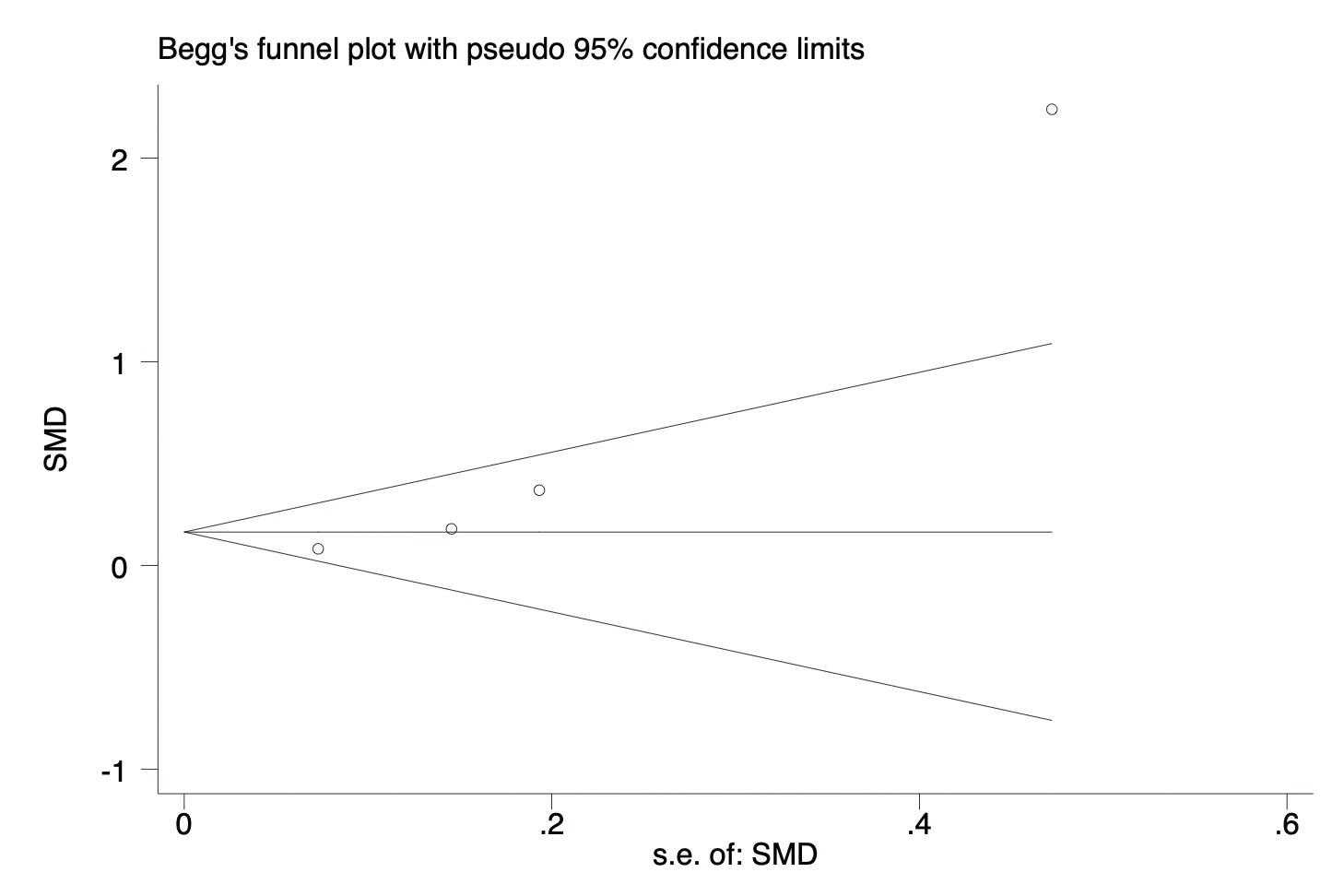

Supplement: Supplemental Information 5 [file peerj-13-19588-s005.tif]
