# Supplementary material for: The impact of multidomain interventions on cognitive and physical function in older adults with subjective cognitive decline: a meta‑analysis and systematic review
Source: PeerJ. 2025 Jun 26;13:e19588. doi: 10.7717/peerj.19588 (PMC12206402; doi:10.7717/peerj.19588)
Supplement: Supplemental Information 8 [file peerj-13-19588-s008.docx]

**The rationale for conducting the systematic review / meta-analysis**

With the global aging population, the decline in cognitive function among older adults, particularly those with subjective cognitive decline (SCD), has become a significant public health issue. SCD is considered an early stage of cognitive decline and a precursor to potential progression to mild cognitive impairment (MCI) or dementia. Existing non-pharmacological interventions, including nutrition, exercise, and cognitive interventions, have shown potential in improving cognitive and physical health in older adults. However, these studies vary in methodology, intervention types, and outcome measures, leading to inconsistent results.

Although some retrospective studies and meta-analyses have explored the impact of multidomain interventions on cognitive decline, many of these studies included different populations or mixed intervention components, which may introduce significant heterogeneity and limit the generalizability of the results. Furthermore, the effects of multidomain interventions specifically targeting SCD patients have not been adequately examined. Therefore, this meta-analysis aims to synthesize the existing evidence, with a particular focus on the impact of multidomain interventions on cognitive and physical functions in older adults with SCD. By employing strict inclusion criteria for high-quality randomized controlled trials (RCTs), this study will ensure more reliable and effective conclusions.

**The contribution to knowledge**

Although some meta-analyses and systematic reviews have reported positive effects of multidomain interventions on cognitive decline, the conclusions of these studies exhibit significant heterogeneity due to differences in intervention components, study designs, and target populations. This meta-analysis will be the first to focus specifically on SCD patients, rigorously selecting high-quality RCTs to provide clearer and more reliable evidence regarding the effects of multidomain interventions. Additionally, this study will compare the effects of multidomain interventions with single-domain interventions, offering direction for future research and promoting the development of more standardized and targeted intervention strategies.

Through this study, the academic community will gain a better understanding of the specific effects of multidomain interventions on cognitive functions in older adults, particularly in areas such as executive function and memory. It will further validate the effectiveness of these interventions in clinical practice. The results will provide crucial insights for public health strategies, helping to develop targeted prevention and intervention measures aimed at delaying the progression of SCD to MCI or dementia.
